# The role of lysophosphatidic acid metabolism in castration-resistant prostate cancer progression

**DOI:** 10.3389/fonc.2026.1842091

**Published:** 2026-05-22

**Authors:** Jinpeng Feng, Jiale Liu, Yi He

**Affiliations:** 1 Jiaxing University Master Degree Cultivation Base, Zhejiang Chinese Medical University, Hangzhou, China; 2 Department of Urology, The Afffliated Hospital of Jiaxing University, Jiaxing, China

**Keywords:** castration resistance, lysophosphatidic acid, metabolic reprogramming, prostate cancer, targeted therapy

## Abstract

Prostate cancer (PCa) is a leading malignancy in men, with mortality primarily attributed to progression to castration-resistant prostate cancer (CRPC). Although androgen deprivation therapy (ADT) initially demonstrates efficacy, most advanced patients eventually develop CRPC—a stage characterized by limited treatment options and poor prognosis. Elucidating the molecular mechanisms underlying CRPC transformation therefore represents a research priority. Tumor metabolic reprogramming has emerged as a hallmark of cancer, and among various metabolic pathways, lysophosphatidic acid (LPA)—a bioactive lipid mediator—has been increasingly implicated in PCa progression, particularly CRPC transformation. This review systematically examines LPA metabolic sources and signal transduction mechanisms and explores how LPA promotes CRPC progression through driving proliferation, survival, invasion, therapy resistance, and tumor microenvironment remodeling. Finally, we discuss the potential and challenges of targeting the LPA signaling pathway as a novel therapeutic strategy for CRPC.

## 1 Introduction

Prostate cancer (PCa) ranks among the most incident and lethal malignancies in men globally, representing the second leading cause of solid tumor-related mortality in Europe and the United States (1). According to GLOBOCAN data, over 1.4 million new PCa cases and approximately 370,000 cancer-related deaths occur annually worldwide (2). Incidence continues rising with population aging and lifestyle changes, while developing countries show increasing diagnosis rates paralleling improved living conditions and medical technology access. Currently, 90% of patients with incurable PCa initially respond to first-line therapies (including ADT and antiandrogen drugs); however, nearly all develop resistance to castration and androgen receptor (AR) inhibitors (e.g., enzalutamide) within 12–33 months (3). Disease progression from hormone-dependent to hormone-independent states markedly increases treatment difficulty. Clinically, PCa progressing after complete androgen blockade is defined as castration-resistant prostate cancer (CRPC) (1, 4). CRPC closely associates with metastasis and rapid progression, typically exhibiting enhanced invasiveness and metastatic potential that sharply deteriorate prognosis.

CRPC transformation mechanisms are complex and multifaceted, involving sustained AR pathway activation (5, 6) [through AR mutations, amplification, or variants like AR-V7 that confer ADT insensitivity (7)], bypass signaling pathway activation (e.g., PI3K/AKT, Wnt/β-catenin), DNA repair defects, and tumor microenvironment (TME) alterations (8). Beyond these classical mechanisms, metabolic reprogramming—an emerging cancer hallmark—provides novel perspectives for understanding CRPC.

Lysophosphatidic acid (LPA), a simple-structured glycerophospholipid, functions as a multifunctional bioactive mediator with diverse biological roles (9). LPA participates in central nervous system development and angiogenesis (10–12), while also contributing to neurodegenerative diseases (10), neuropathic pain (13), inflammatory lung diseases (14), and cancer (15). Through specific G protein-coupled receptors (GPCRs), LPA regulates fundamental cellular processes including proliferation, migration, survival, and differentiation (16). In oncology, LPA demonstrates elevated expression in multiple malignancies including ovarian and breast cancer, promoting malignant progression (17). Growing evidence (18) indicates that in PCa, the LPA metabolic pathway serves as a critical hub connecting intrinsic tumor cell changes with external microenvironmental stimuli, functioning as both “molecular trigger” and “sustained driver” in CRPC transformation. Specifically, LPA engages LPAR1 and LPAR3 to activate PI3K/AKT and RhoA/ROCK signaling, which sustains prostate cancer cell proliferation and survival despite androgen deprivation, while also suppressing autophagy and enhancing invasive capacity—key drivers of castration resistance (19, 20). Abnormalities in LPA production and signal transduction associate with PCa occurrence, development, and metastasis. This review comprehensively summarizes research progress on LPA’s role in PCa progression, from basic LPA biology to specific mechanisms of action, clinical translational value, and targeted therapy prospects, providing researchers with a clear knowledge framework to advance diagnostic and therapeutic strategies targeting this important signaling axis.

## 2 Fundamentals of lysophosphatidic acid metabolism and signal transduction

### 2.1 LPA synthesis and degradation

**Figure 1:**
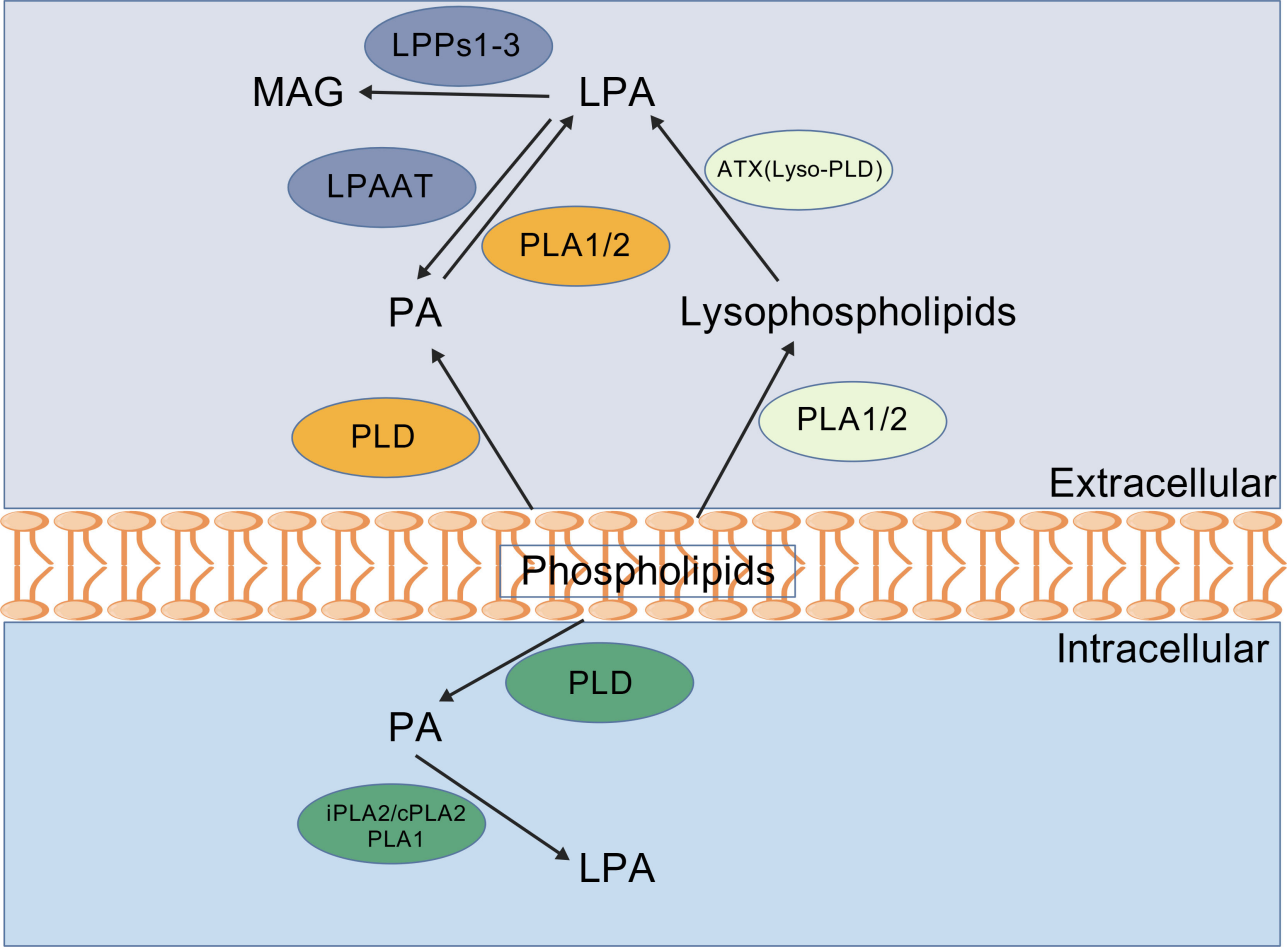
Overview of lysophosphatidic acid (LPA) metabolic pathways. Extracellular LPA is generated from membrane phospholipids via phospholipase D (PLD) followed by phospholipase A1/2 (PLA1/2), or from lysophospholipids via autotaxin (ATX, also named lysoPLD). Intracellular LPA is produced from phosphatidic acid (PA) through PLA1/2 or from PLD- generated PA. Intracellular LPA can be converted back to PA by LPA acyltransferase (LPAAT) or degraded to monoacylglycerol (MAG) by lipid phosphate phosphatases (LPP1-3). iPLA2, calcium- independent phospholipase A2; cPLA2, cytosolic phospholipase A2. The figure was created with BioGDP.com under an academic license.

LPA is a structurally simple bioactive phospholipid, consisting of a glycerol backbone, a phosphate head, and a single acyl chain (21, 22). However, LPA exists as a mixture of several fatty acids *in vivo* condition: unsaturated fatty acids (16:1, 18:1, 18:2 and 20:4) and saturated fatty acids (16:0, 18:0) (23). These variations affect solubility, receptor binding, and biological activity. In human plasma, the most abundant LPA forms are 16:0, 18:2 and 18:1 (24) and in prostate cancer cells, the most abundant forms is 18:1 (25). Multiple cell types—including platelets, fibroblasts, cancer cells, and adipocytes—produce and release LPA via intracellular and extracellular pathways (18) (**Figure 1**). Extracellular LPA is generated through two main routes: membrane phospholipids are converted to phosphatidic acid (PA) by phospholipase D (PLD), then to LPA by phospholipase A2 (PLA2); membrane phospholipids are hydrolyzed to lysophospholipids by PLA2, then converted to LPA by autotaxin (ATX), the key enzyme encoded by ectonucleotide pyrophosphatase/phosphodiesterase 2 (ENPP2) (26, 27). Intracellular LPA is produced from PA hydrolysis by phospholipase A1 (PLA1) or secretory PLA2 (sPLA2) (23, 25, 28). Extracellular LPA binds to LPA receptors (LPARs) to regulate signaling, while intracellular LPA serves as a precursor for glyceroglycolipid synthesis. LPA degradation primarily involves lipid phosphate phosphatases 1-3 (LPPs1-3), converting LPA to monoacylglycerol (29). Additional catabolic enzymes include LPA phosphatase type 6 (acid phosphatase 6/ACP6) and lysophosphatidic acid phosphatase-like protein 6 (PACPL1) (18). Notably, LPA acyltransferase (LPAAT) can reverse metabolic direction by converting LPA back to PA through re-esterification (30). The ATX-LPA-LPAR-LPP axis maintains LPA homeostasis critical for normal physiology (31). Dysregulation through altered expression or activity of PLA1/2, ATX, LPA, LPAR or LPP contributes to numerous diseases including cancer, cardiovascular disorders, autoimmune conditions, neurological diseases, and inflammatory pathologies (32).

### 2.2 The LPA-LPAR signaling pathway

LPA’s diverse biological functions have earned it the designation “multifunctional phosphate messenger.” Since the first LPA receptor identification and cloning in 1996 (33), six specific LPA receptors (LPAR1-6) have been discovered, classified into two families (34, 35) (**Table 1**). LPARs can couple with various heterotrimeric G proteins, including Gαs, Gαi, Gαq, Gα12 and Gα13. The specific interaction between LPARs and the Gα and Gβγ subunits is the core factor regulating downstream signaling pathways and cellular responses. Consequently, the direction of LPA-LPAR signaling is primarily determined by the specific G protein subtypes coupled to the involved LPARs, which in turn drive downstream signaling cascades and ultimately regulate various cellular biological behaviors, including proliferation, survival, inflammatory responses, immune responses, migration, and differentiation. Specifically, activation of Gαs by LPARs stimulates adenylyl cyclase and increases cyclic AMP (cAMP) levels; activation of Gαi inhibits adenylyl cyclase activity (36); activation of Gαq initiates phospholipase C (PLC), which generates inositol triphosphate (IP3) and diacylglycerol (DAG) (37); while Gα12 and Gα13 can regulate Rho family GTPases, ultimately affecting the actin cytoskeleton and cell motility (38).

**Table 1:** LPA receptors.

Family	Receptors	Alternative names
EDG family	LPAR1, LPAR2, LPAR3	EDG2, EDG4, EDG7
Purinergic GPCR family	LPAR4, LPAR5, LPAR6	P2Y9/GPR23, GPR92, P2Y5

Each LPAR subtype exerts distinct effects on cancer cell behavior through specific downstream pathways. LPAR1 couples with Gαi/o, Gαq/11, and Gα12/13 to activate AKT, MAPK, PLC, and Rho pathways, regulating proliferation, migration, and cytoskeletal dynamics; it is implicated in ovarian, gastric, osteosarcoma, and nasopharyngeal cancers (34). LPAR1 is a key mediator of LPA (18:1)-induced proliferation and migration in prostate cancer cells. In PC-3 and DU145 cells, LPAR1 activates ERK1/2 and p38α via Gi/o, promoting migration (39). LPAR1 also facilitates androgen-independent growth by enhancing AR nuclear localization (40). In 3D organotypic culture, LPAR1 signaling through Gα12/13 and RhoA stabilizes acinar morphogenesis and suppresses invasion, suggesting a context-dependent metastasis-suppressor role (41). LPAR2 binds Gα12/13, Gαq/11, and Gαi/o to trigger PI3K, PLC, MAPK, Rac, Rho, and Ras signaling (9); highly expressed in testes and leukocytes, its aberrant expression in malignancies suggests a role in tumor progression (42, 43). In prostate cancer, its expression is relatively low, but it can heterodimerize with CD97 to amplify Rho-dependent invasion (44). LPAR3 shows tissue-specific expression (high in heart, testis, prostate, pancreas; low in lung, ovary, brain) and couples with Gαq/11 and Gαi/o (but not Gα12/13) to initiate MAPK and PLC cascades, with abnormal expression noted in some tumors (45). LPAR3 is upregulated in prostate cancer epithelia compared to benign glands (46). It promotes invasion, lymphangiogenesis, and glycolysis-induced radioresistance. Specifically, LPAR3 (together with LPAR1) mediates ROS production via PLC/PKC/NADPH oxidase, leading to VEGF-C expression and lymphatic metastasis (47, 48). Circular RNA derived from LPAR3 (circLPAR3) sponges miR-513b-5p to upregulate JPT1, enhancing glycolysis and reducing radiosensitivity (49). In platelet-PCa cell cocultures, LPAR3 overexpression is associated with poor patient survival (50). Among Purinergic GPCR family, LPAR4 is widely expressed and uniquely couples with four G proteins—Gαi/o, Gαq/11, Gα12/13, and Gαs (the latter being atypical for LPARs) (45, 51). In pancreatic cancer, isolation stress or chemotherapy downregulates miR-139-5p, which releases the suppression of LPAR4 expression. Upregulated LPAR4 then activates the AKT/CREB signaling axis in an LPA (18:1)-independent manner, driving cell-autonomous production of a fibronectin-rich extracellular matrix that promotes tumor initiation, stress tolerance, and drug resistance (52). However, its role in PCa remains unexplored. LPAR5 interacts with Gα12/13, Gαi/o, and Gαq/11 to induce stress fiber formation via Gα12/13 and elevate intracellular Ca^2+^ through Gαq/11; its knockout impairs LPA-induced pancreatic cancer cell migration (53). Besides, Its high expression in prostate cancer correlates with poor prognosis, and it contributes to apoptotic resistance in platelet-induced PCa cell surviva (50). LPAR6, abundantly expressed in epithelia and hair follicles, is linked to hair disorders when mutated (54); although its role in migration remains unclear, silencing LPAR6 enhances tumor cell drug sensitivity, suggesting therapeutic potential (55). Evidence from research suggests that LPAR6 is upregulated in androgen-independent prostate cancer cells and promotes metastasis. LSD1 demethylase represses LPAR6 transcription (56). It is also a potential therapeutic target in metastatic PCa.

### 2.3 Non-canonical LPA receptors

In recent years, several additional transmembrane proteins have been identified as non-canonical LPA receptors, including the GPCRs GPR87, GPR35, and P2Y10, as well as the ion channel TRPV1 (57–60). These receptors expand the signaling repertoire of LPA beyond the classical LPAR family. Additionally, peroxisome proliferator-activated receptorγ(PPARγ) functions as an intracellular LPA receptor (61). Beyond the six classic LPARs, several other transmembrane proteins and a nuclear receptor have been identified as functional LPA receptors. GPR87 is a GPCR with high affinity for LPA (unspecified species) (EC_50_≈36 nM), coupling to Gαq to mobilize intracellular Ca^2+^. It is expressed in human prostate, as well as placenta, testis, brain, and skeletal muscle (57). GPR87 is involved in p53-dependent survival after DNA damage, but its specific role in prostate cancer progression has not been fully elucidated. GPR35 selectively recognizes 2-acyl LPA species (e.g., 2-oleoyl LPA) and activates Ca^2+^ signaling, RhoA, and ERK (58). It is highly expressed in gastrointestinal and lymphoid tissues (62). Recent evidence indicates that GPR35 overexpression is correlated with poor prognosis and advanced clinical stage in prostate cancer, making it a potential prognostic biomarker (63). P2Y10 functions as a dual receptor for both LPA (18:1) and sphingosine-1-phosphate (S1P), activating Gαq-mediated Ca^2+^ signals. It is expressed in reproductive organs, brain, lung, and skeletal muscle (59). Its role in prostate cancer remains unknown. TRPV1 is a non-GPCR ion channel that directly binds LPA (18:1) at its C-terminal Lys710 residue (K_0_≈754 nM), leading to channel opening and pain behavior independent of G proteins (60). TRPV1 is expressed in prostate cancer cells and has been implicated in proliferation and apoptosis regulation; LPA (18:1) may thus signal through TRPV1 in the prostate tumor microenvironment (64). PPARγis an intracellular nuclear receptor that directly binds LPA (18:1) and functions as a transcription factor (61). In prostate cancer, PPARγ activation can modulate lipid metabolism, inflammation, and cell differentiation (65). Although LPA-PPARγ signaling has been studied in various contexts, its direct contribution to CRPC progression remains to be explored. Collectively, these non-canonical receptors expand the LPA signaling network and offer new potential targets for prostate cancer therapy.

**Figure 2:**
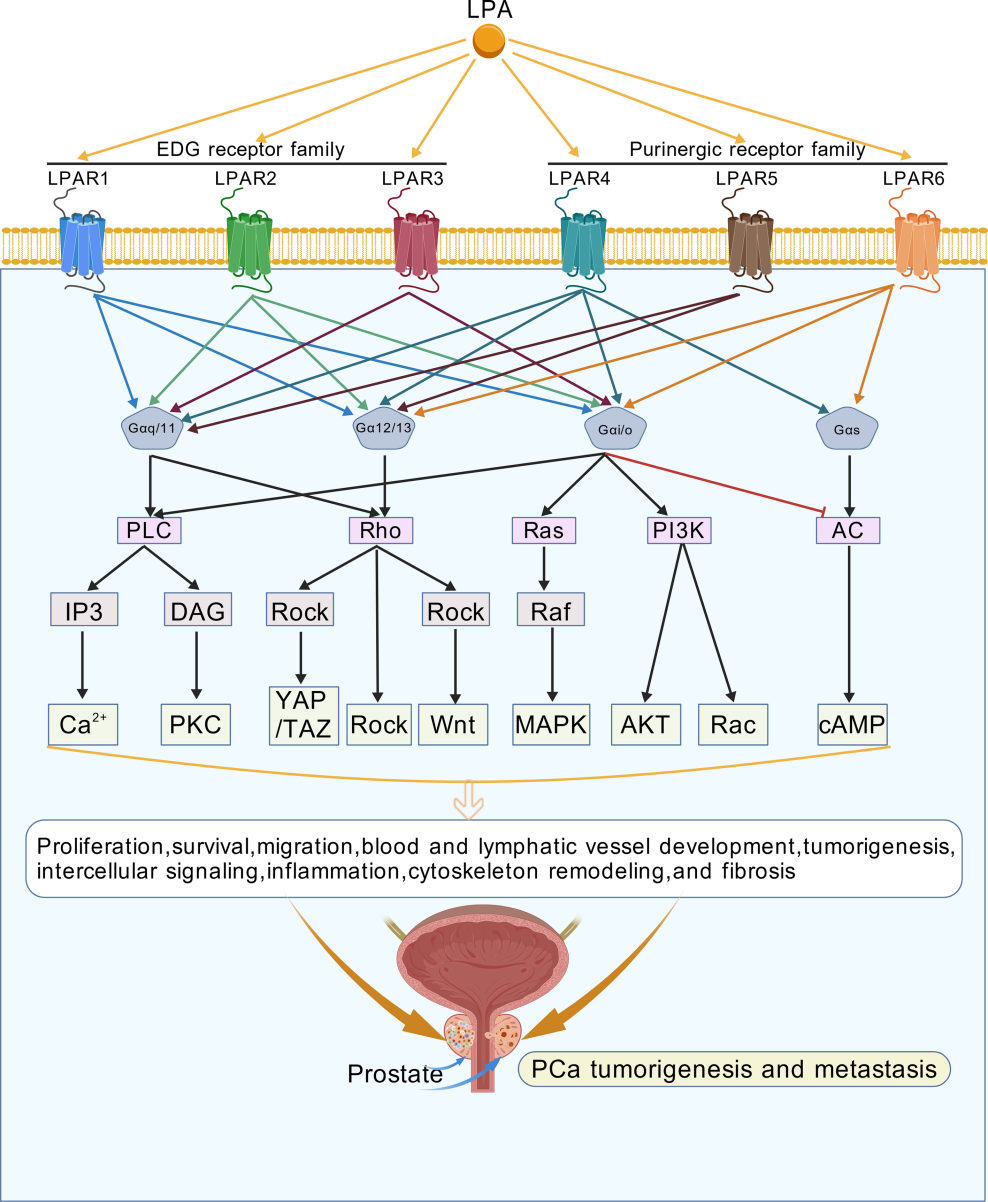
LPA receptor signaling pathways. LPA binds to six G protein- coupled receptors (LPAR1-6), which are classified into the EDG family (LPAR1-3) and the purinergic receptor family (LPAR4-6). These receptors couple to distinct G proteins (Gαq/11, Gα12/13, Gαi/o, Gαs) and activate downstream effectors including phospholipase C (PLC), Rho, Ras, PI3K, and adenylyl cyclase (AC). This leads to diverse cellular responses such as proliferation, survival, migration, cytoskeletal remodeling, inflammation, and tumorigenesis, with specific relevance to prostate cancer (PCa). The figure was created with BioGDP.com under an academic license.

Not all LPA receptors recognize all LPA species equally. LPA differ in acyl chain length (e.g., 16:0, 18:0, 18:1, 20:4) and degree of saturation, and these structural variations influence receptor binding and biological activity. For instance, LPAR3 and LPAR6 exhibit a preference for 2-acyl LPA over 1-acyl LPA, whereas LPAR1, LPAR2, LPAR4, and LPAR5 show broader selectivity. Moreover, specific LPA species such as 18:1 (oleoyl)-LPA, 18:2 (linoleoyl)-LPA, and 20:4 (arachidonoyl)-LPA may differentially activate downstream signaling pathways depending on the receptor repertoire of the target cell (9). These differences in ligand-receptor selectivity have important implications for understanding LPA signaling in prostate cancer, where the expression profile of LPARs is altered during disease progression. This receptor diversity underscores the complexity of LPA signaling and its context-dependent roles in prostate cancer progression (**Figure 2**).

## 3 Dysregulated LPA signaling in prostate cancer

During PCa progression, key LPA metabolic pathway components exhibit significant dysregulation. Zeng et al. found that compared to benign prostate glands, PCa epithelial cells show significantly decreased LPAR1 mRNA but increased LPAR3 mRNA, suggesting a receptor expression “switch” potentially associated with disease progression and androgen independence (46). Nouh et al. subsequently confirmed that LPA synthases ATX and AGK show absent or low expression in non-tumor prostate epithelial cells but frequent expression in high-grade prostatic intraepithelial neoplasia and PCa tissues (66). Importantly, high ATX expression significantly associates with a higher primary Gleason grade (≥4), capsular invasion, and postoperative prostate-specific antigen (PSA) failure risk, highlighting its role in malignant progression (66). Epigenetic regulation of ENPP2 also participates: Panagopoulou et al. found that increased methylation of ENPP2 promoter-associated CpG sites in PCa and other tumors correlates with downregulated mRNA expression and poor prognostic parameters (67). Lipidomic analyses have identified LPA as one of several lipid species with potential prognostic value in various malignancies, although direct validation in prostate cancer-specific cohorts remains warranted (68).

Beyond these descriptive alterations, emerging evidence has begun to uncover the upstream mechanisms driving the dysregulation of LPARs and LPA-producing enzymes in prostate cancer. For instance, the histone demethylase LSD1 (lysine-specific demethylase 1) directly represses transcription of LPAR6; depletion of LSD1 leads to increased LPAR6 expression, which promotes paxillin phosphorylation and enhances migration and invasion of androgen-independent PCa cells (56). This establishes an epigenetic mechanism for LPAR6 upregulation in metastatic prostate cancer. DNA methylation also plays a role: hypermethylation of the ENPP2 promoter is associated with decreased ATX expression and poor prognosis in prostate adenocarcinoma (67), indicating that both transcriptional and post-transcriptional epigenetic events contribute to LPA metabolic dysregulation. Although direct evidence linking classic oncogenes such as MYC or PTEN to LPAR or ATX/AGK expression is still limited, indirect observations suggest potential connections. The androgen receptor (AR) signaling axis may influence the expression profile of LPA (unspecified species)-producing enzymes: androgen-sensitive LNCaP cells predominantly express AGK, whereas androgen-insensitive PC-3 and DU145 cells express ATX (46). Moreover, PTEN loss, a common event in CRPC, enhances dependency on p110β-mediated LPA (unspecified species) signaling in a mouse model of prostate tumorigenesis (69), raising the possibility that PTEN deficiency might indirectly augment the LPA pathway, though direct transcriptional regulation has not been demonstrated. Collectively, these findings indicate that both genetic and epigenetic events—including LSD1-mediated repression, DNA methylation, and potentially AR-dependent transcriptional programs—contribute to the aberrant LPA signaling observed during CRPC transformation. Further studies are warranted to fully elucidate how prostate cancer oncogenes orchestrate the LPA metabolic network.

These findings collectively depict continuous LPA metabolic pathway activation during PCa progression, particularly during transition to invasive phenotypes.

## 4 Core mechanisms of LPA in prostate cancer progression

### 4.1 Driving androgen-independent proliferation and survival

A core CRPC feature is sustained tumor cell proliferation and survival in low-androgen environments, with LPA signaling providing key alternative growth signals. Guo et al. (40)directly demonstrated LPAR’s importance: LPAR1 expresses in LPA (18:1)-responsive, androgen-insensitive PCa cells but not in androgen-dependent LNCaP cells (unresponsive to LPA’s proliferative effects). Stable LPAR1 expression in LNCaP cells not only conferred responsiveness to LPA-induced proliferation but also significantly accelerated *in vivo* tumor growth. Importantly, LPAR1-transduced LPA signaling promotes AR nuclear localization, with proliferative effects inhibitable by the antiandrogen bicalutamide, indicating LPA-AR pathway synergy—partially explaining traditional AR-targeted therapy failure in CRPC.

LPA also promotes survival by inhibiting apoptosis and autophagy. Garofano et al. (50) revealed platelet-cancer cell bidirectional crosstalk: platelets confer apoptosis resistance through LPA (unspecified species)-LPAR signaling, independent of AR blockade. Among involved signaling molecules, LPAR3 and LPAR5 overexpression associates with poor patient survival. Regarding autophagy—an important nutritional stress survival mechanism—Genc et al. (70) confirmed that in PC-3, LNCaP, and Du145 cells, LPA (18:1) activates ERK and AKT-mTOR pathways, phosphorylating S6K and ULK, thereby inhibiting autophagy marker LC3 maturation and autophagosome formation. Chang et al. also reported LPA (18:1) inhibits serum deprivation-induced autophagy in PC-3 cells (71). In androgen-deprived microenvironments, LPA may help cancer cells maintain energy homeostasis and avoid apoptosis by inhibiting excessive autophagy, conferring survival advantage.

LPA-induced proliferation and survival involve multiple kinase pathways. LPA (18:1) activates AKT and IκBα through LPAR1 and LPAR3, regulating RhoA and NF-κB activity—molecules that jointly promote cell cycle progression and apoptosis resistance (19). In DU-145 cells, LPA (18:1) triggers transient ERK and AKT phosphorylation, inhibitable by FFA4 receptor agonists (e.g., TUG-891), indicating lipid receptor cross-regulation (72). Additionally, Park et al. (73) showed in PC-3 cells that LPA (18:1) pretreatment inhibits ionomycin-induced focal adhesion kinase (FAK) and p130CAS degradation, reducing cell detachment—an effect dependent on LPA receptors and paxillin phosphorylation. These findings collectively demonstrate LPA supports PCa cell proliferation and survival through complex mechanistic networks.

### 4.2 Association with oxidative stress and metabolic reprogramming

Tumor cell metabolic reprogramming underlies malignant phenotypes, with LPA signaling closely linked to this process. In advanced prostate cancer, the expression of LPARs is increased and signals are mediated through the G12 family (e.g., Gα13). While Gα13’s oncogenic role in migration and invasion is characterized, its mitochondrial and oxidative stress functions remain unclear. GPCR-Gα13 signaling reduces superoxide levels, and constitutively active Gα13 overexpression promotes antioxidant gene activation. In human samples, mitochondrial superoxide dismutase 2 associates with PCa risk and Gleason score, suggesting LPA signaling may affect tumor progression through redox balance regulation (74).

Lin et al. (75) directly demonstrated in PC-3 cells that LPA (18:1) induces rapid mitochondrial reactive oxygen species (ROS) production through LPAR1 and LPAR3 via phospholipase C/protein kinase C/NADPH oxidase pathway activation. Klomsiri et al. (76) further localized LPA (18:1)-induced ROS production to “redoxosomes”—early endosomes internalizing LPAR1 and containing NADPH oxidase components. These active endosomes are sites of protein cysteine sulfenic acid modification, involving signaling molecules including AKT2 and PTP1B, thus linking LPA signaling to redox-sensitive transduction. LPA signaling also associates with glycolysis activation—the Warburg effect core. Chen et al. (49) found circular RNA circLPAR3 upregulates JPT1 expression by sponging miR-513b-5p, promoting glycolysis and reducing radiosensitivity in PCa cells. Huang et al. (20) showed high glucose induces VEGF-C expression through LPAR1/3-AKT-ROS-LEDGF signaling, simultaneously enhancing aerobic glycolysis. These findings suggest LPA signaling provides metabolic adaptability for CRPC cells under therapeutic stress by driving glycolysis and regulating oxidative stress.

### 4.3 Promoting invasion, metastasis, and therapy resistance

Invasion and metastasis represent lethal CRPC features, with LPA functioning as a potent driver through multiple signaling pathways and cellular structure remodeling. Hwang et al. (19, 77) systematically demonstrated LPA (18:1) activates RhoA and NF-κB through AKT/IκBα signaling, promoting functional invadopodia formation and enhancing PC-3 cell invasive capacity. This LPA-RhoA-NF-κB axis also proves crucial for *in vivo* tumor growth and osteolytic lesions. LPA plays key roles in inducing lymphangiogenesis and angiogenesis—the basis of lymphatic and hematogenous metastasis. Lin et al. (48) confirmed LPA (18:1) upregulates VEGF-C expression in PC-3 cells through LPAR1/3-ROS-LEDGF-dependent pathways. Wu and Lee’s review further emphasized the LPA-VEGF-C axis’s core role in regulating tumor lymphangiogenesis and lymph node metastasis (78). Subsequent research revealed calreticulin’s mediating role: LPA (18:1) promotes CRT expression through LPAR1/3-ROS-eIF2α pathways, with CRT knockdown inhibiting VEGF-C induction and *in vivo* lymphatic vessel density (47). Beyond VEGF-C, LPA (18:1) induces VEGF-A expression; however, the aryl hydrocarbon receptor can inhibit LPA-induced VEGF-A expression by competing with HIF-1α for their common dimerization partner ARNT, revealing complex TME regulatory networks (79).

Regarding therapy resistance, beyond aforementioned apoptosis resistance and metabolic adaptability, LPA signaling directly affects radiotherapy and drug therapy efficacy. Chen et al.’s study on the circLPAR3/miR-513b-5p/JPT1 axis promoting glycolysis and radioresistance provides direct evidence for LPA pathway involvement in radioresistance (49). Additionally, Härmä et al. (41) found in three-dimensional organotypic models that LPA (unspecified species) and LPAR1 promote PCa cell epithelial maturation and block spontaneous invasion through Gα12/13 signaling, suggesting LPA signaling has context-dependent metastasis-inhibiting functions—dysregulation of which may lead to invasive phenotype emergence. This context-dependent dual role highlights LPA signaling complexity.

## 5 Therapeutic strategies targeting the LPA signaling axis

As a key lipid signaling molecule, LPA promotes CRPC transformation by activating multiple pro-tumor pathways. Developing inhibitors targeting LPA metabolic enzymes or receptors has become an important research direction for overcoming CRPC.

### 5.1 Inhibition of LPA metabolic enzymes

ATX inhibition represents a priority strategy, as ATX is the main extracellular LPA source. Kitakaze et al. (80) developed a high-throughput activity assay for glycerophosphodiester phosphodiesterase 4 (GDE4) and GDE7 using fluorescent substrate FS-3, finding that some ATX inhibitors also inhibit these intracellular LPA synthases—laying foundation for selective inhibitor development. Studies using clinical-stage ATX inhibitor IOA-289 in pancreatic cancer models revealed ATX’s complex TME role: IOA-289 reduces connective tissue growth factor secretion in cancer-associated fibroblasts by regulating LPA/LPAR signaling. Interestingly, ATX inhibition leads to increased extracellular LPA levels, suggesting ATX may regulate LPA-receptor interactions through LPA chaperone functions beyond enzymatic activity—providing new mechanistic insights for ATX inhibitor efficacy (81). Beyond these preclinical findings, IOA-289 has entered clinical development. A first-in-human, randomized, double-blind, placebo-controlled phase I study in healthy volunteers (clinical trial ID: NCT05027568) demonstrated that oral IOA-289 was well tolerated with no clinically significant adverse events. Plasma exposure increased dose-proportionally, and the drug produced a dose-dependent reduction of circulating LPA (especially LPA 18:2), with an estimated IC_50_ of 15 ng/mL for LPA inhibition. These data confirmed target engagement and supported further clinical evaluation (82). Based on these results, a phase Ib trial of IOA-289 in combination with standard-of-care chemotherapy is currently ongoing in patients with metastatic pancreatic cancer (clinical trial ID: NCT05586516). Thus, IOA-289 represents the most advanced ATX-targeting agent in clinical oncology, and its development provides a strong rationale for evaluating ATX inhibition as a novel therapeutic strategy for CRPC. Pundalik et al. (83) reported corosolic acid effectively inhibits secretory phospholipase A2IIa activity, reducing LPA (unspecified species) precursor supply, demonstrating anti-tumor activity in PC-3 cells and Ehrlich ascites carcinoma models. Clinical data also support targeting ATX: Nakamura et al. found serum ATX levels decrease in PCa patients after surgery, suggesting ATX may associate with tumor burden or nutritional status, serving as efficacy monitoring markers (84, 85). In other disease models (idiopathic pulmonary fibrosis, ischemic myocardial injury), ATX inhibitor PF-8380 effectively alleviates tissue fibrosis, inflammatory responses, and endothelial dysfunction (86–88). These successes across different pathological contexts provide strong theoretical support for ATX inhibitor application in PCa treatment.

### 5.2 Blockade of LPA receptor signaling

Directly targeting LPA receptors—particularly subtypes highly expressed or functionally critical in PCa—represents another core therapeutic strategy. LPAR1 and LPAR3 are primary targets. The LPAR1/3 antagonist Ki16425 has been proven in multiple studies to effectively inhibit LPA-induced cell proliferation, migration, VEGF-C expression, and *in vivo* lymphatic metastasis (47, 89, 90). David et al. also identified heparin-binding EGF-like growth factor as an LPAR1 activation biomarker, useful for monitoring LPAR1-targeted therapy pharmacodynamics in clinical trials (91). Other LPAR subtypes are also under investigation. A breast cancer study (92)found LPAR3 antagonists inhibit LPA (18:1)-induced cancer stem cell expansion through LPAR3 and TRPC3 calcium channels—providing new target ideas for PCa, particularly therapy-resistant subtypes. Ketscher et al. (56) pointed out that in androgen-independent PCa cells, LPAR6 upregulation associates with metastasis, suggesting LPAR6 as another potential therapeutic target.

Emerging strategies include bifunctional inhibitors simultaneously targeting ATX and LPARs. The brominated phospholipid analog BrP-LPA reportedly inhibits both ATX activity and multiple LPARs, demonstrating excellent effects in protecting blood-brain barrier integrity and improving mitochondrial function in ischemic stroke models (88). This “one stone two birds” strategy may more effectively suppress abnormally active LPA signaling, particularly suitable for complex environments with redundant and compensatory signaling pathways like PCa.

### 5.3 Other emerging strategies to target the LPA axis

Beyond ATX/LPAR inhibition, other LPA-targeting strategies have emerged, including neutralizing extracellular LPA, blocking alternative LPA-producing enzymes, and enhancing LPA degradation.

Monoclonal antibodies (e.g., Lpathomab) sequester LPA and has demonstrated neuroprotective effects in central nervous system (CNS) injury models by neutralizing extracellular LPA (18:1) (93), but it also shows significant cross-reactivity with more abundant lipids such as LPC and PA (94). Although not yet tested in PCa, this strategy could be particularly relevant for CRPC, given the elevated LPA levels in the tumor microenvironment and the redundant receptor expression that often limits the efficacy of single-receptor antagonists.

Targeting other LPA-producing enzymes offers another avenue. While ATX is the primary extracellular source, intracellular LPA production is mediated by other enzymes. Inhibitors of secretory phospholipase A2 (sPLA2), which generates LPA (unspecified species) precursors, have shown preclinical activity. For instance, corosolic acid inhibits sPLA2IIa activity and reduces PC-3 prostate cancer cell viability (83). Similarly, acylglycerol kinase (AGK) is upregulated in PCa and contributes to LPA (18:1) production, and its downregulation has been shown to inhibit PCa cell proliferation (66, 95).

Enhancing LPA degradation is a less explored but mechanistically appealing strategy. The primary route for LPA clearance is dephosphorylation by lipid phosphate phosphatases (LPPs). Restoring LPP isoform balance—by inhibiting pro-tumorigenic LPP2 or increasing tumor-suppressive LPP1/LPP3—suppresses tumor progression; For instance, LPP2 knockout reduces c-Myc and breast tumor growth (96). Given that LPP activity is often reduced in aggressive tumors, LPP-targeted modulation represents a future direction for CRPC therapy.

### 5.4 Combination therapy strategies

Due to signaling pathway redundancy, monotherapy targeting LPA metabolism may have limited efficacy, positioning combination therapy as a critical direction. Accumulating evidence implicates the LPA signaling axis in therapy resistance across multiple cancers. In non-small cell lung cancer, acquired resistance to third-generation EGFR-TKIs closely correlates with LPA metabolic reprogramming. LPA (unspecified species) promotes epithelial-mesenchymal transition and migration while attenuating EGFR-TKI efficacy, effects that are reversible by disrupting LPA-LPAR signaling (97). This offers a direct reference for prostate cancer (PCa): in patients resistant to AR pathway inhibitors (e.g., abiraterone, enzalutamide), the LPA axis may serve as an analogous “escape pathway.” By activating non-classical AR signaling, LPA (unspecified species) drives castration-resistant PCa (CRPC); thus, combining LPA pathway inhibitors with enzalutamide or abiraterone may synergistically suppress AR signaling and overcome castration resistance. Additional rationale for combination strategies derives from LPA’s profound impact on the tumor microenvironment (TME). LPA (unspecified species) modulates immune cell function, promotes angiogenesis and fibrosis, and fosters an immunosuppressive TME that supports tumor growth (98, 99). In ovarian cancer, LPA drives progression and chemoresistance via upregulation of the long non-coding RNA UCA1 (100). Hence, combining LPA axis inhibitors with immune checkpoint blockade could reverse immunosuppression, elicit anti-tumor immunity, and yield synergistic effects. Given the close link between LPA signaling and Hippo pathway effectors YAP/TAZ (LPA (unspecified species) activates YAP/TAZ), combining LPAR antagonists with emerging YAP/TAZ inhibitors may prove effective in highly malignant PCa (101, 102). Furthermore, LPA (unspecified species) suppresses T cell activity within the TME; combining ATX inhibitors with PD-1/PD-L1 blockade may restore anti-tumor immune responses and inhibit PCa progression. In ER-positive breast cancer, tamoxifen resistance is associated with activation of the LPC-LPA phospholipid synthesis axis due to Tafazzin deficiency, and targeting this pathway restores tamoxifen sensitivity (103). Collectively, these findings strongly suggest that combining LPA axis inhibitors with AR-targeted therapies or chemotherapeutic agents may represent effective sensitization strategies in PCa.

**Figure 3:**
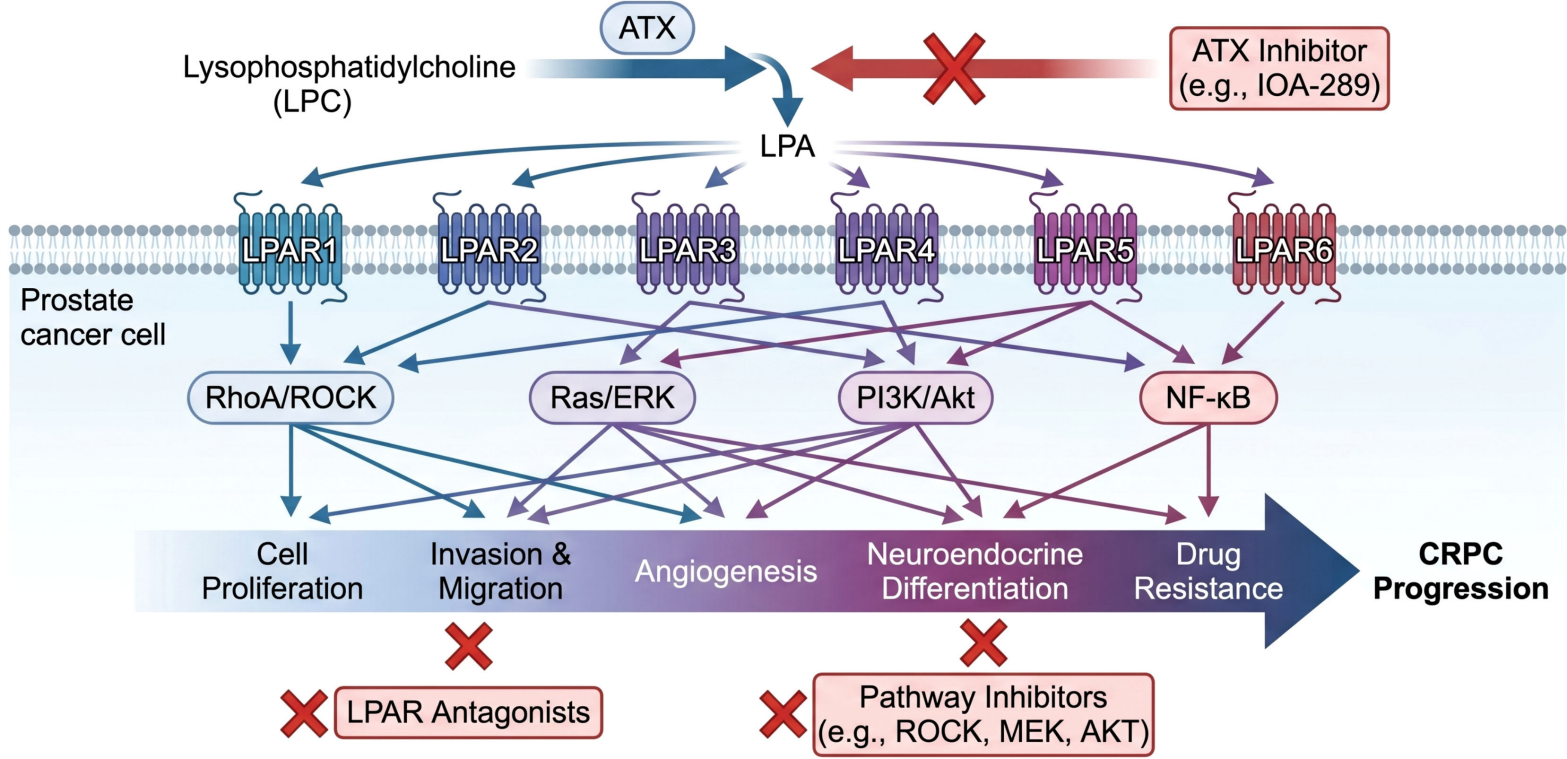
Therapeutic targeting of the LPA-ATX axis in castration- resistant prostate cancer (CRPC). Extracellular LPA is produced from lysophosphatidylcholine (LPC) by ATX and acts via LPAR1-6 to activate downstream signaling pathways (RhoA/ROCK, Ras/ERK, PI3K/AKT, NF-κB), which drive CRPC progression through cell proliferation, invasion, angiogenesis, neuroendocrine differentiation, and drug resistance. Potential therapeutic strategies include ATX inhibitors (e.g., IOA-289), LPAR antagonists, and pathway inhibitors targeting ROCK, MEK, or AKT. The figure was created with BioGDP.com under an academic license.

Although most of these strategies are preclinical, they offer a rich pipeline for CRPC therapy, warranting systematic evaluation in PCa models (**Figure 3**).

## 6 Discussion

The LPA metabolic pathway is deeply involved in CRPC transformation through complex mechanistic networks. This network includes synthase (ATX, AGK) upregulation and epigenetic dysregulation, dynamic receptor expression profile changes (LPAR1, LPAR3, LPAR6), and sustained activation of multiple downstream signaling pathways (Gα12/13-RhoA-ROCK, PI3K-AKT-mTOR, ERK, NF-κB). Biological consequences include induction of androgen-independent proliferation, apoptosis resistance, autophagy inhibition, metabolic reprogramming (glycolysis activation), oxidative stress regulation, epithelial-mesenchymal transition, invadopodia formation, lymphangiogenesis, and ultimately therapy resistance and metastasis.

While preclinical findings are encouraging, translating LPA-targeted strategies into clinical practice faces several hurdles. Several key questions remain to be addressed: Is LPA pathway activation a driver or a bystander in CRPC transformation? How can functional redundancy among LPARs be overcome for precise therapeutic targeting? Can circulating LPA species serve as liquid biopsy biomarkers for early detection of CRPC or prediction of treatment response?

Future research should prioritize: integrating single-cell sequencing with spatial transcriptomics to map LPA-related networks in CRPC; using patient-derived organoids and microfluidic models to track LPA signaling plasticity and identify therapeutic nodes; establishing LPA metabolic risk signatures via multi-omics for patient stratification; combining epigenetic and metabolic flux analyses to uncover LPA-driven metabolic reprogramming; and evaluating LPA inhibitor combinations with AR blockade, immunotherapy, and metabolic interventions in preclinical models, followed by rapid clinical translation through micro-dose or umbrella trials. In summary, a deeper understanding of the complex role of the LPA metabolic network in CRPC will pave new avenues for overcoming therapy resistance and bring renewed hope to patients.
